# Chronic Calcaneal Osteomyelitis Due to a Retained Coral Fragment: An Unusual Cause of Heel Pain

**DOI:** 10.5334/jbsr.4208

**Published:** 2026-01-27

**Authors:** Eimantas Abelkis, Loes Schiphouwer

**Affiliations:** 1 Department of Radiology, HAGA Teaching Hospital and Haaglanden Medical Center, The Hague, Netherlands; 2 Department of Radiology, Haaglanden Medical Center, The Hague, Netherlands

**Keywords:** osteomyelitis, penumbra sign, foreign body, coral

## Abstract

We report a case of calcaneal osteomyelitis caused by a retained foreign body—a coral fragment. The patient presented with nonspecific heel pain 12 years after the initial injury. MRI revealed an intraosseous lesion demonstrating the ‘penumbra’ sign, consistent with chronic osteomyelitis and abscess formation. CT demonstrated a foreign body embedded within the abscess cavity. Surgical exploration confirmed the presence of a coral fragment.

*Teaching point:* Retained foreign bodies in bone can remain asymptomatic for many years, eventually presenting with signs of osteomyelitis.

## Introduction

Foreign bodies in the soft tissues of the foot are common in clinical practice; however, retained objects in bone are rare. If not removed, these may eventually cause osteomyelitis. We describe an unusual case of chronic osteomyelitis caused by a retained coral fragment and presenting 12 years after the initial trauma. To the best of our knowledge, this is the first reported case of osteomyelitis resulting from a coral injury.

## Case Report

A 35-year-old female presented to our institution complaining of heel pain for one year that worsened in the last two months. She tried physiotherapy, non-steroidal anti-inflammatory drugs and heel cushions to alleviate the pain; however, there was no improvement. Physical examination revealed a normal foot arch and a painless range of motion of the ankle joints. There was localized pressure pain at the level of the posterior tibial tendon. A clinical diagnosis of posterior tibial tendinitis was made.

**Figure 1:**
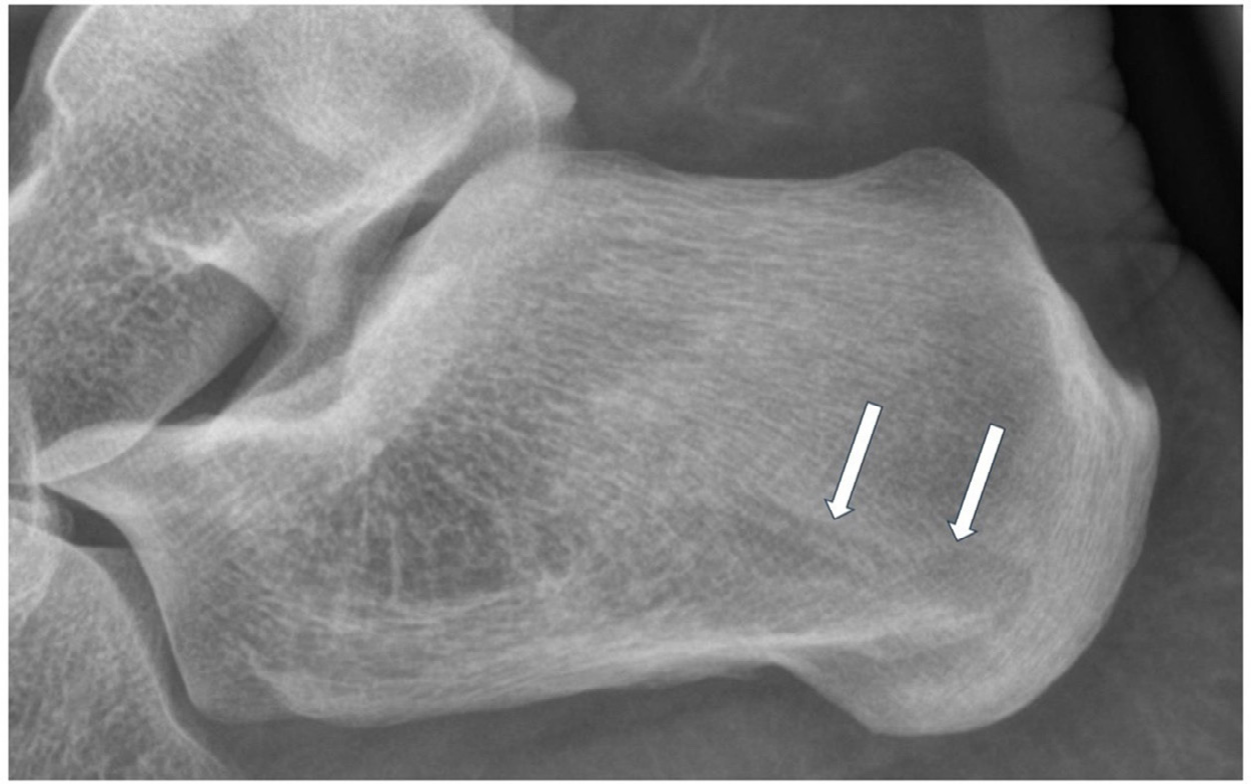
Arrows show a subtle osteolytic lesion with a narrow zone of transition in the calcaneus.

A lateral radiograph of the calcaneus was obtained to assess the presence of a heel spur or other bone abnormalities. The initial assessment revealed no abnormalities. However, retrospective analysis revealed an osteolytic lesion in the posterior calcaneus (Figure 1).

**Figure 2:**
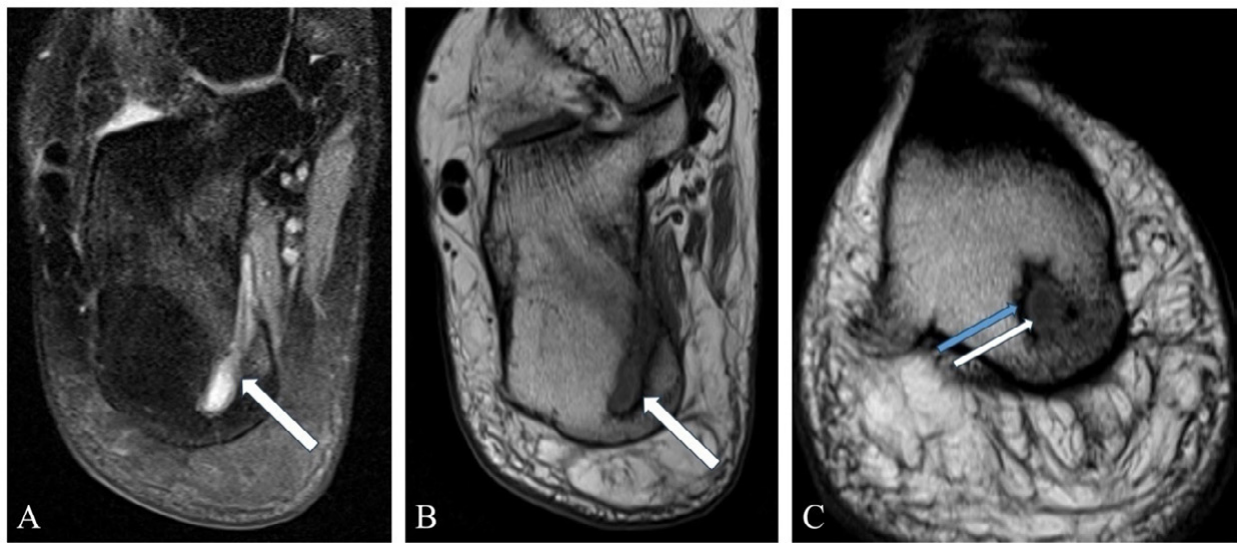
A lesion in the posteromedial calcaneus with a hyperintense signal on T2W-FS **(A)** and a hypointense signal on T1W **(B)** sequences, consistent with an abscess. Note bone marrow oedema surrounding the lesion and cortical breakthrough. **(C)** T1W image demonstrating the ‘penumbra’ sign (white arrow) and reactive sclerosis (blue arrow).

Subsequently, an MRI of the ankle was performed to evaluate suspected tendinopathy. The posterior tibial tendon showed no abnormalities. However, there was a well-circumscribed lesion located in the posteromedial calcaneus (Figure 2). On the T1-weighted sequence, the lesion showed low signal intensity centrally, surrounded by a thin hyperintense rim, consistent with the ‘penumbra’ sign. Along the outermost margin, reactive sclerosis was present. On the medial side, there was cortical breakthrough with a small soft tissue extension. Based on the radiological characteristics, a diagnosis of chronic osteomyelitis with an intraosseous abscess was made. Additionally, within the described lesion, a thin, elongated structure was observed, showing low signal intensity on all sequences. The differential diagnosis for this structure included sequestrum, dystrophic calcification, and a foreign body.

**Figure 3:**
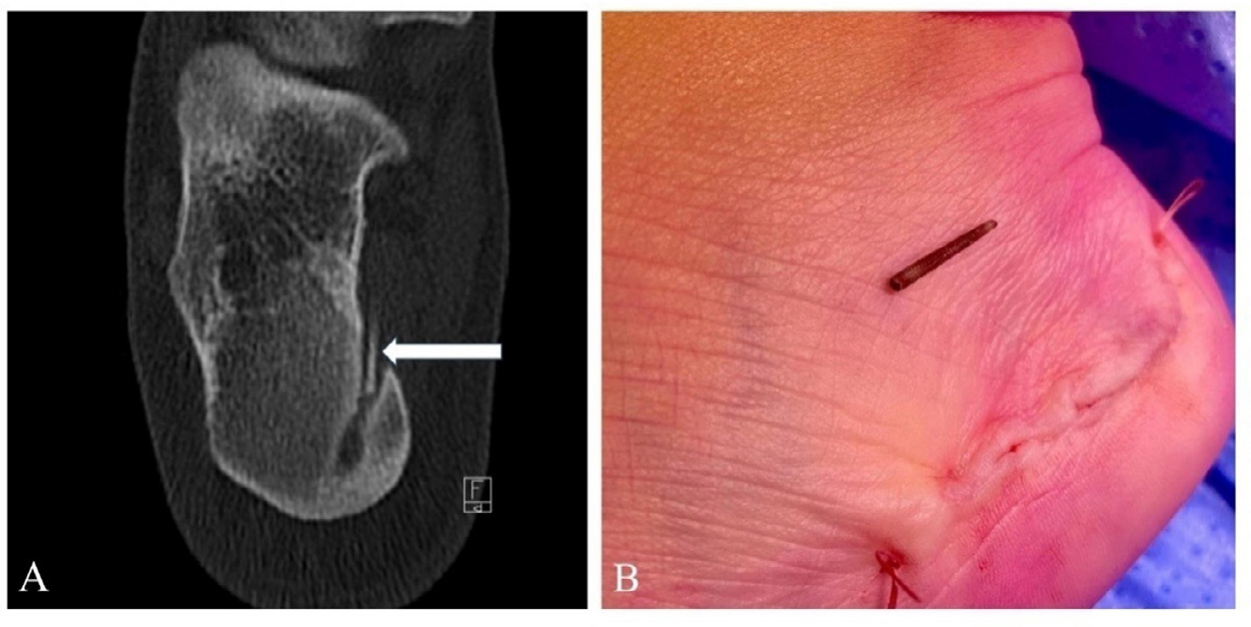
**(A)** CT scan demonstrates a linear structure within the abscess cavity extending into the soft tissues. **(B)** Intraoperative findings confirmed the presence of a coral fragment.

The patient was referred for a CT-guided biopsy to obtain samples for microbiological analysis. The planning CT during the procedure showed a sharp, elongated foreign body in the abscess cavity (Figure 3A). During the procedure, the patient disclosed that 12 years earlier, while in Mexico, she stepped on coral while snorkelling. At the time, the wound was cleaned and sutured at a local hospital. Following the accident, she had not experienced any symptoms until the recent episode.

Subsequently, under ultrasound guidance, a wire localizer was inserted to mark the abscess. The abscess was surgically evacuated, and the foreign body removed. Intraoperatively, the presence of a coral fragment was confirmed (Figure 3B). At the one-year follow-up, the patient reported no pain or signs of infection.

## Discussion

Osteomyelitis still poses a diagnostic challenge. Systemic signs, such as fever, may not be present, and laboratory tests may be normal [1]. Moreover, on imaging, osteomyelitis can mimic a spectrum of other entities, including Langerhans cell histiocytosis, chondroblastoma and Ewing sarcoma [2, 3].

The ‘penumbra’ sign can be helpful in making the diagnosis of subacute and chronic osteomyelitis on imaging. It is seen on unenhanced T1-weighted MRI sequences and represents a hyperintense rim surrounding the hypointense lesion [4]. This hyperintensity corresponds to vascularized granulation tissue lining the abscess cavity. On the outer margin, there is usually sclerosis and bone marrow oedema. The specificity of the ‘penumbra’ sign is reported to be 96% and sensitivity –27% [4]. This sign is not pathognomonic for osteomyelitis; it has been reported in Langerhans cell histiocytosis and Ewing sarcoma [2, 3].

Our literature search identified only one other case involving coral injury to the bone by Palmanovich et al. [5]. They described a hallux injury with aseptic osteolysis; microbiology and tissue specimens were negative for bacteria.

Penetrating foot wounds with foreign bodies in the soft tissues are not uncommon in clinical practice. However, bone injury, and specifically osteomyelitis, due to retained foreign bodies, is rare. According to the study by Vidyadhara et al., which described four cases of foot osteomyelitis caused by thorn pricks, these accidents usually occur while walking barefoot [6].

Interestingly, retained foreign bodies can remain silent for many years before presenting with osteomyelitis. Rogoff et al. described a case of calcaneal osteomyelitis manifesting 25 years after an initial puncture wound of the foot by a chicken bone [1]. Moreover, Surov et al. reported a case of osteomyelitis of the leg due to retained grenade splinters presenting 60 years after an injury during the Second World War [7]. Late presentation was also seen in our case.

## Conclusion

Foreign bodies retained in soft tissues or bone can lead to osteomyelitis even many years after the initial injury. Our case underscores the importance of a thorough clinical history and suggests that even remote, relevant traumatic accidents should be documented. Furthermore, the ‘penumbra’ sign shows high specificity for the diagnosis of subacute and chronic osteomyelitis; however, it is not pathognomonic.
